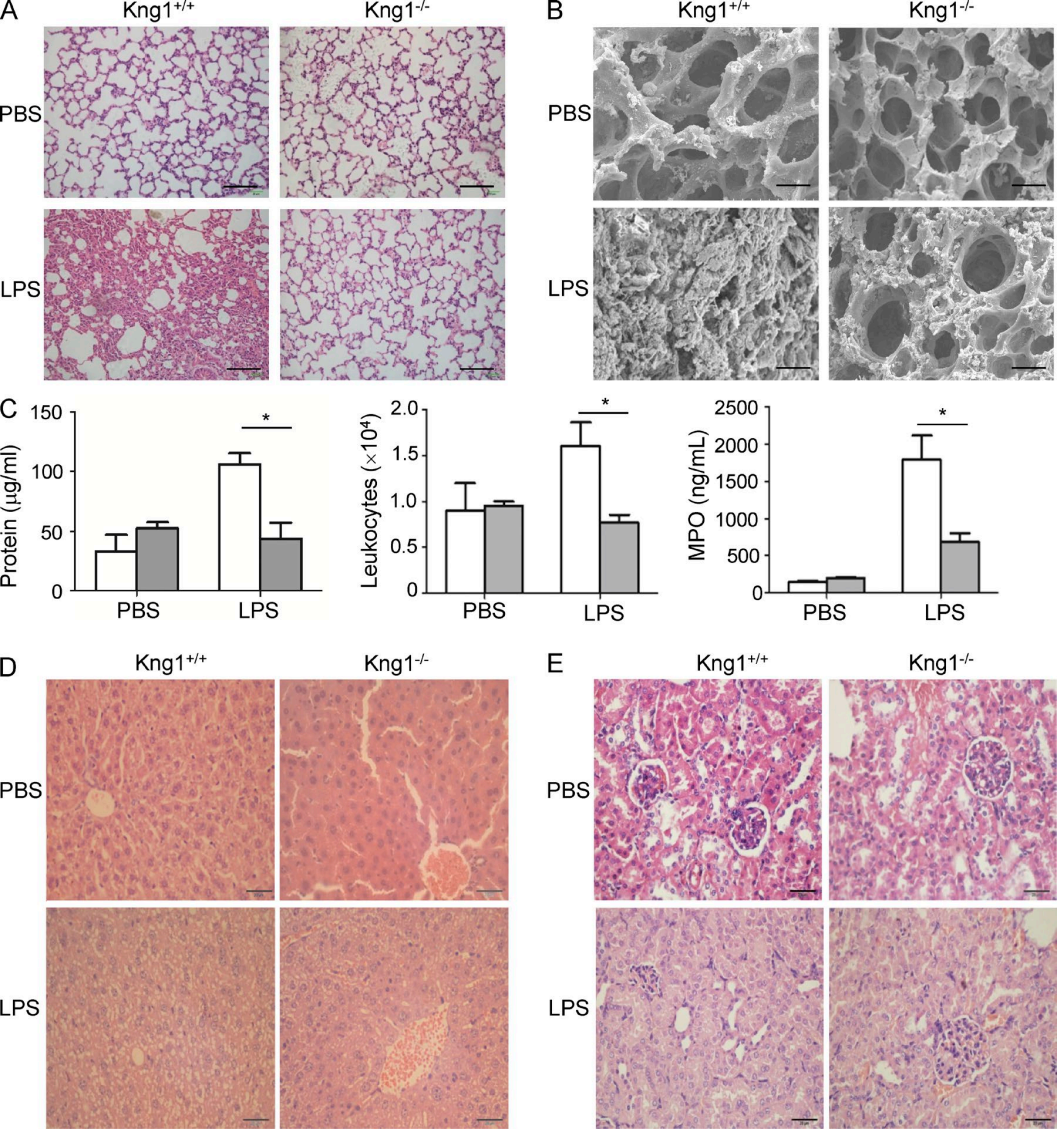# Correction: An essential role of high-molecular-weight kininogen in endotoxemia

**DOI:** 10.1084/jem.2016190012072018c

**Published:** 2019-01-07

**Authors:** Aizhen Yang, Zhanli Xie, Bo Wang, Robert W. Colman, Jihong Dai, Yi Wu

Vol. 214, No. 9, September 4, 2017. 10.1084/jem.20161900

The authors regret that an error appeared in the original version of Fig. 3. In the upper panels of D, the image for Kng1^+/+^ mice was taken from the Kng1^–/–^ mouse sample. The corrected figure appears below.

The online HTML and PDF versions of this paper have been corrected. The error remains only in the print version.

**Figure:**